# Care of the Sick

**Published:** 1860-06

**Authors:** 


Care of the Sick.—Not to allow a patient to be waked, intentionally or accidentally, is the *sine qua non* of all good nursing. If he is roused out of his first sleep, he is almost certain to have no more sleep. It is a curious, but quite intelligible fact, that if a patient is waked, after a few hours instead of a few minutes sleep, he is much more likely to sleep again. Because pain, like irritability of brain, perpetuates and intensifies itself. If you have gained a respite of either in sleep, you have gained more than the mere respite. Both the probability of recurrence and of the same intensity will be diminished; whereas both will be terribly increased by want of sleep. This is the reason why a patient, waked in the early part of his sleep, loses not only his sleep, but his early power to sleep. A healthy person who allows himself to sleep during the day, will lose his sleep at night; but it is exactly the reverse with the sick generally; the better will they be able to sleep.—Miss Nightingale.

